# Diet and irritable bowel syndrome: an update from a UK consensus meeting

**DOI:** 10.1186/s12916-022-02496-w

**Published:** 2022-09-13

**Authors:** A. Rej, A. Avery, I. Aziz, C. J. Black, R. K. Bowyer, R. L. Buckle, L. Seamark, C. C. Shaw, J. Thompson, N. Trott, M. Williams, D. S. Sanders

**Affiliations:** 1 Academic Unit of Gastroenterology, Royal Hallamshire Hospital, Sheffield Teaching Hospital NHS Foundation Trust, Sheffield, UK; 2 Division of Nutritional Sciences, School of Biosciences, University of Nottingham, Nottingham, UK; 3 Leeds Gastroenterology Institute, St James’s University Hospital, Leeds, UK; Leeds Institute of Medical Research at St James’s, University of Leeds, Leeds, UK; 4 Department of Nutrition and Dietetics, Royal United Hospitals NHS Foundation Trust, Bath, UK; 5 Specialist Gastroenterology Community Dietetic Service, Somerset Partnership NHS Foundation Trust, Bridgwater, UK; 6 Information Manager/Specialist Gastroenterology Dietitian, Guts UK Charity, 3 St Andrews Place, London, NW1 4LB UK

**Keywords:** Irritable bowel syndrome, Low FODMAP diet, Gluten-free diet, Traditional dietary advice

## Abstract

There has been a renewed interest in the role of dietary therapies to manage irritable bowel syndrome (IBS), with diet high on the agenda for patients. Currently, interest has focussed on the use of traditional dietary advice (TDA), a gluten-free diet (GFD) and the low FODMAP diet (LFD). A consensus meeting was held to assess the role of these dietary therapies in IBS, in Sheffield, United Kingdom.

Evidence for TDA is from case control studies and clinical experience. Randomised controlled trials (RCT) have demonstrated the benefit of soluble fibre in IBS. No studies have assessed TDA in comparison to a habitual or sham diet. There have been a number of RCTs demonstrating the efficacy of a GFD at short-term follow-up, with a lack of long-term outcomes. Whilst gluten may lead to symptom generation in IBS, other components of wheat may also play an important role, with recent interest in the role of fructans, wheat germ agglutinins, as well as alpha amylase trypsin inhibitors. There is good evidence for the use of a LFD at short-term follow-up, with emerging evidence demonstrating its efficacy at long-term follow-up. There is overlap between the LFD and GFD with IBS patients self-initiating gluten or wheat reduction as part of their LFD. Currently, there is a lack of evidence to suggest superiority of one diet over another, although TDA is more acceptable to patients.

In view of this evidence, our consensus group recommends that dietary therapies for IBS should be offered by dietitians who first assess dietary triggers and then tailor the intervention according to patient choice. Given the lack of dietetic services, novel approaches such as employing group clinics and online webinars may maximise capacity and accessibility for patients. Further research is also required to assess the comparative efficacy of dietary therapies to other management strategies available to manage IBS.

## Background

Irritable bowel syndrome (IBS) is a common disorder of gut-brain interaction, with a reported global prevalence of around 4% using the Rome IV criteria [1]. The burden of IBS on patients is considerable and the costs to healthcare systems and society are substantial, driving the need for effective interventions [2]. Although there are many pharmacological options for the management of IBS, dietary management remains high on the agenda for the majority of patients [3]. A recent study highlighted that the most preferred treatment for individuals with IBS was dietary therapy (48%), followed by pharmacotherapy (29%) and psychotherapy (22%) [4]. In addition, 63 to 84% of people with IBS report symptoms related to eating specific food items, with a greater number of food triggers identified by individuals with more severe IBS [5, 6].

Research into dietary therapies in IBS has focussed on traditional dietary advice (TDA), a gluten-free diet (GFD) and the low FODMAP diet (LFD). Current guidelines vary globally in terms of recommendations for implementation of dietary therapies in IBS. British guidelines suggesting the use of TDA followed by the LFD for the dietary management of IBS, whereas American and Canadian guidelines suggest the use of the LFD, with no mention of TDA [7–10]. In addition, no guidelines recommend the use of a GFD due to insufficient evidence. However, further research has been published since the conception of these guidelines, and the aim of our UK consensus meeting was, therefore, to provide a meaningful framework for UK dietetic practice with respect to IBS management.

## Format for consensus meeting

A consensus meeting between gastroenterologists and dietitians with a specialist interest in IBS was held in Sheffield, United Kingdom (UK) in June 2021. PubMed was searched to identify relevant studies pertaining to dietary therapies in IBS, using the MeSH terms diet, dietary therapies, irritable bowel syndrome, low FODMAP, gluten free and traditional diet to identify relevant articles. Existing literature was reviewed. Following the meeting, the literature was re-reviewed to assess for any updates until March 2022, with a consensus being made on the current evidence for dietary therapies in IBS.

## Traditional dietary advice

TDA for patients with IBS consists of dietary and lifestyle management [7]. This encompasses practical measures such as reducing alcohol intake, reduction of caffeine intake, avoidance of spicy meals, reduction in fat intake and increase in fluid intake as well as alteration of fibre intake [7]. TDA reduces dietary triggers for people with IBS including some that are not FODMAP containing, which is an important consideration. With the exception of dietary fibre, the evidence base for recommending these dietary changes relies on case-control studies and clinical experience, rather than robust randomised controlled trials (RCTs). Although RCTs have demonstrated the benefit of fibre in IBS, this appears to be limited to soluble fibre, such as ispaghula, rather than insoluble fibre, like wheat bran [8]. To date, there have been no RCTs assessing the efficacy of TDA in comparison to a habitual diet or sham diet.

## Gluten-free diet

There has been an exponential growth over the last decade in the use of a GFD outside the context of coeliac disease (CD) [11]. The prevalence of self-reported wheat sensitivity has been reported at approximately 10% globally [12]. Wheat appears to be a key trigger for symptoms in patients with IBS, reported at between 23 and 49% of individuals [5, 13–15].

In view of this, the role of a GFD has been explored in IBS. There have been a number of RCTs demonstrating its efficacy, with key studies outlined in Table 1. The majority of RCTs exploring the GFD in IBS have demonstrated its efficacy at short-term follow-up, between 4 and 6 weeks [16–20]. It is worth noting that a study by Biesiekierski et al. failed to show dose dependent effects of gluten after individuals were placed on a LFD [21]. However, this may have been due to the study design, with individuals having an anticipatory nocebo response to the intervention. There have also been non-randomised trials also demonstrating the efficacy of the GFD. A prospective study in 41 patients with IBS-D demonstrated that 71% had a clinical response, defined as drop in IBS Symptom Severity Score [IBS-SSS] of ≥ 50 points, 6 weeks after implementation of a GFD [22]. Interestingly, 72% of individuals with a clinical response planned to continue the diet in the long term, with symptom reduction, anthropometric and biochemical features (body mass index, haemoglobin, ferritin, folate, vitamin B12 and albumin) being maintained at 18 months [22]. Nevertheless, there remains a lack of data demonstrating the long-term efficacy of a GFD for IBS and further research is required.

**Table 1:** Key studies evaluating the GFD in IBS

Lead author	Year	Study design	Study duration	Total number of participants	Outcome
Biesiekierski [16]	2011	DBPC trial	6 weeks	34 IBS patients (Rome III)	68% had inadequate control of symptoms with gluten compared to 40% with placebo (*p* = 0.0001)
Biesiekierski [21]	2013	Crossover DBPC trial	9 weeks	37 IBS patients (Rome III)	No dose dependent effects of gluten seen when placed on a diet low in FODMAPs
Vasquez-Roque [17]	2013	RCT	4 weeks	45 IBS-D patients (Rome II)	Individuals on gluten containing diet had more bowel movements per day compared to those on gluten-free diet (*p* = 0.04)
Aziz [22]	2015	Prospective study	6 weeks	41 IBS-D patients (Rome III)	71% had clinical response to GFD
Shahbazkhani [18]	2015	DBPC trial	6 weeks	72 IBS patients (Rome III)	Symptom improvement in gluten containing group lower than placebo (26% vs 84%, *p* < 0.001)
Zanwar [19]	2015	DBPC trial	4 weeks	60 IBS patients (Rome III)	Higher overall symptom VAS score with gluten vs placebo (week 4; 25 vs 10, *p* < 0.05)
Barmeyer [14]	2017	Prospective study	4 months	35 IBS-D/M patients (Rome III)	34% of patients noted to be responders to GFD
Paduano [23]	2019	Prospective study	4 weeks	42 IBS patients (Rome IV)	Reduction in symptom severity (*p* < 0.01), bloating (*p* < 0.01), abdominal pain (*p* < 0.01) on GFD
Pinto-Sanchez [24]	2021	Prospective study	4 weeks	50 IBS patients (Rome III)	75% clinical response for individuals on GFD with positive antigliadin antibodies, 38% response for those without
Rej [20]	2022	RCT	4 weeks	101 IBS patients (Rome IV)	58% clinical response to GFD

Total number of studies; *n* = 10, total number of participants; *n* = 517

In terms of predictors of responsiveness to a GFD, a prospective study of 50 patients with IBS demonstrated that individuals with antigliadin IgG and IgA reported less diarrhoea than those without antibodies (*p* = 0.03), using the Birmingham IBS symptom questionnaire [24]. Whilst this highlighted that antigliadin antibodies could potentially be used as a predictor for response to a GFD in IBS, the prevalence of antigliadin antibody positivity in the study was 50%, markedly higher than the 21% reported in the validation cohort [24]. Further studies are required to assess whether antigliadin antibodies are a potential biomarker for predicting response to a GFD in IBS. Interestingly, in this study, individuals with some gluten exposure on the GFD still had a clinical response [24]. Similar improvements in overall GI symptoms were seen amongst individuals with strict compliance and minimal transgressions on the GFD (*Z* difference − 3.5 minimal transgression (*p* < 0.001) vs − 3.2 (*p* = 0.001) strict compliance in IBS AGA positive) [24]. This suggests that individuals with IBS may not need to follow the strict GFD used for treating patients with coeliac disease. The threshold of gluten reduction for patients with IBS to derive symptom benefit is still unclear. This is supported by a recent RCT, which demonstrated that 58% of individuals on a GFD, where there may have been some level of cross contamination, still had a clinical response, defined as a reduction in IBS-SSS of ≥ 50 points [20].

Mean dietary intake of fructans in the UK has been reported at 4.0 g daily [25]. Although wheat contains relatively low quantities of fructans, the high frequency of bread consumption has led to wheat being a major contributor to fructan consumption in the UK, with 66% of daily fructan intake being from wheat [25, 26]. Therefore, many individuals who adopt a GFD are likely to reduce their fructan intake substantially [27]. Although the benefits of a GFD in IBS have been demonstrated, it has been postulated that this may be due to a reduction in fructans (FODMAPs) rather than gluten. This is supported by a double-blind crossover challenge in 59 individuals who self-administered a GFD, who were randomly assigned to diets containing gluten, fructans or placebo [28]. This study demonstrated significantly higher Gastrointestinal Symptom Rating Scale (GSRS) scores for individuals consuming fructans rather than gluten (*p* = 0.049) [28]. In addition, a recent double-blind placebo RCT in 110 patients with IBS demonstrated that consumption of FODMAPs led to modest increases in IBS-SSS compared to the consumption of gluten (240 vs 208, *p* = 0.013) [29]. However, a recent study in patients with IBS showed no correlation of GI symptoms with fructan intake [30].

Whilst fructan intake has been explored as a key component of wheat in symptom generation, several other components may also trigger symptoms in IBS. Gluten itself may trigger symptoms, with a gluten containing diet showing an association with higher small bowel permeability, as well as a decrease in the expression of tight junction proteins [17, 31]. In addition, several other components of wheat may also play a role in the pathophysiology, with alpha-1 amylase trypsin inhibitors (ATIs) and wheat germ agglutinins (WGAs) also been postulated as triggers [32, 33].

Potential nutritional concerns remain with implementation of a GFD. This data is extrapolated from individuals with CD. In terms of macronutrient intake, fat intake appears to be increased on a GFD, with some studies showing a high consumption of saturated fatty acids potentially increasing the risk of cardiovascular disease [34]. In addition, individuals may have a reduced carbohydrate and fibre intake [34]. Micronutrient deficiencies of iron, calcium and magnesium have also been noted [34]. There is little data on the nutritional adequacy of a GFD in IBS. A recent RCT in IBS, where patients received a GFD, demonstrated no changes in macronutrient intake following a GFD, but a reduction in micronutrient intake of magnesium and thiamine was noted [20]. However, it is worth noting that this study did not employ the strict GFD employed in CD [20].

The impact of a GFD on the gut microbiome in patients with IBS is currently unclear. In healthy individuals, a GFD appears to affect bacterial populations, such as a decreasing pro-inflammatory bacteria such as *Veillonellaceae* [35, 36]. Reduction in beneficial gut populations such as *Bifidobacterium* have also been noted in healthy individuals on a GFD [37], as well as in CD [36]. There is little data on the effects of a GFD in patients with IBS. A recent study demonstrated a reduction in abundance of *Actinobacteria*, *Parabacteroides johnsonii*, and *Eubacteriumrectale*, as well as *Ruminococcusalbus* and *R. bromii* following a GFD in IBS [20]. Further research is required to elucidate the effect of a GFD on the gut microbiome in patients with IBS.

## Low FODMAP diet

Fermentable oligo-, di-, mono- saccharides and polyols (FODMAPs) are short chain carbohydrates, which are poorly absorbed, increasing small bowel water content by osmosis, and releasing gases, predominantly carbon dioxide and hydrogen, from bacterial fermentation [38]. Both healthy individuals and patients with IBS have similar physiological responses following FODMAP ingestion, as demonstrated by MRI imaging [39]. However, it is likely that individuals with IBS have increased symptoms following FODMAP consumption as a result of visceral hypersensitivity [40].

There has been significant interest in the role of the LFD to manage patients with IBS, particularly over the last decade. The LFD is implemented through a multiphasic approach. The initial phase, which is generally implemented over 4-8 weeks, involves the strict reduction of all FODMAP groups. If individuals have an improvement in symptoms, then the next phase is implemented, over a 6- to 10-week period. FODMAPs are re-introduced, enabling patients to identify specific FODMAP triggers that induce symptoms. Finally, patients enter the long-term personalisation phase, where a less restrictive diet is consumed, and only those FODMAPs which induce symptoms are excluded [41].

The majority of studies assessing the LFD have focussed on the initial short-term elimination phase, with a large number of RCTs demonstrating its efficacy (Table 2). Recent meta-analyses, which pooled outcome data amongst studies that used IBS-SSS to assess clinical response, demonstrated a mean reduction of around 50 points following the LFD [42, 43]. It is worth noting that studies assessing the LFD have had variable comparator diets, such as a sham diet, habitual diet, TDA, Australian diet and high FODMAP diet, highlighting the heterogeneity of studies to date [44].

**Table 2:** Key studies evaluating the LFD in IBS

Lead author	Year	Study design	Study duration	Total number of participants	Outcome
**Short term**					
Staudacher [45]	2012	RCT	4 weeks	41 IBS patients (Rome III)	68% response to LFD vs 23% on habitual diet (*p* = 0.005)
Pedersen [46]	2014	RCT	6 weeks	123 IBS patients (Rome III)	Significant reduction in IBS-SSS on LFD compared to normal Danish/Western diet (133 vs 68, *p* < 0.01)
Halmos [47]	2014	RCT	3 weeks	30 IBS patients (Rome III)	Lower overall gastrointestinal symptom scores on a LFD compared to Australian diet (23 vs 45, *p* < 0.001)
Bohn [48]	2015	RCT	4 weeks	75 IBS patients (Rome III)	50% clinical response to LFD
Eswaran [49]	2016	RCT	4 weeks	92 IBS-D patients (Rome III)	52% reported adequate relief of symptoms
Zahedi [50]	2017	RCT	6 weeks	110 IBS-D patients (Rome III)	Significant reduction in IBS-SSS following LFD (264 vs 108, *p* < 0.001)
Patcharatrakul [51]	2019	RCT	4 weeks	70 IBS patients (Rome III)	Global IBS symptom severity score using VAS were significantly lower following LFD compared to commonly recommended diet (39 vs 54, *p* < 0.01)
Goyal [52]	2021	RCT	16 weeks	101 IBS-D patients (Rome IV)	63% response to LFD at week 4 and 53% response at week 16
**Long term**					
de Roest [53]	2013	Observational	16 months	90 IBS patients (Definition not stated)	Most symptoms including abdominal pain, bloating, flatulence and diarrhoea improved following LFD (*p* < 0.001)
Peters [54]	2016	RCT	6 months	74 IBS patients (Rome III)	82% improvement noted following LFD relative to baseline symptoms at 6 months
O’Keeffe [55]	2017	Prospective	6-18 months	74 IBS patients (Rome III)	57% reported satisfactory relief at long term following LFD
Weynants [56]	2020	Retrospective	100 weeks	90 IBS patients (Rome III)	Patients following the LFD reported less abdominal pain than those who had stopped following the diet (*p* = 0.044)
Bellini [57]	2020	Prospective	6-24 months	73 IBS patients (Rome IV)	Clinical response reported at 83% in those who continued LFD at long term
Rej [58]	2021	Observational	44 months	205 IBS patients (Rome III)	60% reported satisfactory relief at long term following LFD

Total number of studies (short term); *n* = 8, total number of participants; *n* = 642

Total number of studies (long term); *n* = 6, total number of participants; *n* = 606

Currently, there are limited long-term studies demonstrating the efficacy of a low FODMAP diet. A study in 90 patients with IBS, with a mean follow-up of 16 months, demonstrated that symptom improvement was maintained during longer term follow-up, with adherence reported at 76% [53]. Likewise, a RCT in 74 patients with IBS demonstrated sustained symptom response at 6 months in 82% of patients on a LFD [54]. More recently, two studies from the UK have demonstrated ongoing symptom relief with the LFD during long-term follow-up, with adequate relief of symptoms reported in 57% (6–18 months follow-up) and 60% (44 months mean follow-up) of individuals, respectively [55, 58]. In addition, between 65 to 82% of individuals who are following a LFD are in the personalisation phase at long-term follow-up [55, 58, 59].

Interestingly, it has been demonstrated that many individuals are excluding gluten as part of the LFD during long-term follow-up. A recent study noted that 83% of individuals in the personalisation phase of the LFD consume ‘free-from’ products, with gluten or wheat free products being the commonest (68%) [58]. In addition, the commonest dietary requirement when eating out was noted to a gluten or wheat free diet (43%) [58]. This study highlighted those individuals maybe using these diets as part of a LFD due to greater awareness of the GFD [60].

Like the GFD, there are potential nutritional concerns of the LFD. The majority of studies have evaluated the impact of the LFD on nutritional adequacy in the initial strict restriction phase, at short-term follow-up. A RCT in 75 patients with IBS demonstrated that individuals following the LFD had a marked reduction in total carbohydrate and fibre intake at 4 weeks, which was not seen with TDA [48]. Moreover, whilst total energy intake was reduced with both interventions, the reduction was greater with the LFD [48]. Another RCT demonstrated a statistically significant decrease in several micronutrients with a LFD compared to the modified National Institute for Health and Care Excellence (mNICE) diet. However, the differences noted with the LFD only remained for riboflavin when corrected for calorie intake. Consequently, uncertainties remain with regards to the nutritional adequacy of the LFD in the short term [61].

For individuals in the re-introduction phase of the LFD, a recent RCT in 101 patients with IBS-D demonstrated a reduction in energy, carbohydrate, fat and fibre intake at 4 weeks, but a gradual improvement at 16 weeks [52]. During long-term follow-up of 6-18 months in one study and 44 months in another, the LFD has been shown to have similar nutritional adequacy to individuals on a habitual diet (Table 2) [55, 58]. These findings suggest that the LFD may be more nutritionally balanced over the longer term, which may reflect the less restrictive nature of the personalisation phase.

The impact of the LFD on the gut microbiome is unclear currently. A systematic review found no influence of a LFD on overall microbial diversity [42]. *Bifidobacterium* is known to be a key butyrate producer in the colon, playing an important role in colonic health [62]. Most studies looking at the LFD found that the abundance of *Bifidobacteria* and/or the overarching phylum *Actinobacteria* to be reduced [42]. Faecal bacterial profiles have been suggested to potentially predict responsiveness to a LFD. A RCT in 67 patients with IBS demonstrated that responsiveness to a LFD could be predicted by faecal bacterial profiles [63]. However, a more recent RCT, using the same method of stool analysis failed to replicate these findings [20].

Adherence to all phases of the LFD can be challenging. A case series in patients with functional gastrointestinal symptoms who had previously been recommended to follow the LFD demonstrated significantly better dietary adherence with a dietitian-led approach versus other methods (96% vs 71%, *p* = 0.02, short term; 70% vs 39%, *p* = 0.02, re-challenge phase; 65% vs 29%, *p* < 0.01, personalisation phase) [59]. However, as can be seen from this study, adherence to the LFD fell gradually with time, highlighting the challenges of maintaining this diet. Whilst the evidence base for the LFD is derived from dietetic-led LFD studies, the majority of individuals following the LFD do not receive specialist dietetic input. A survey from the United States (US) found that only 21% of gastroenterologists commonly refer patients to registered dietitians [60].

## Challenges to the implementation of dietary therapies in IBS

Whilst the majority of individuals receiving dietetic-led LFD advice appear to be adhering to the personalisation phase of the LFD, adherence falls to only 29% amongst those receiving non dietetic-led advice [59]. Less restrictive approaches have been proposed, including the ‘bottom up’ approach, whereby patients reduce their intake of only a few FODMAPs at initial implementation, based on a full dietary history and patient reported triggers [64]. For many patients this may largely translate into adoption of a GFD and reduction of fructans [27, 65]. However, patients may report food triggers other than wheat and alternative approaches, such as focusing on the reduction of excess fructose and polyols, may also be beneficial [64]. A recent RCT demonstrated total FODMAP intakes of 7.6g/day, 15.2g/day and 22.4g/day for the LFD, TDA and GFD respectively [20]. This highlights the differing FODMAP intakes with these diets, with the GFD (22.4g/day total FODMAPs) and TDA (15.2g/day total FODMAPs) potentially being a form of a ‘bottom-up’ approach to manage patients with IBS [20].

A threshold reduction of 12 g daily of FODMAP intake has been suggested as the threshold required for symptom improvement [59]. Whilst the majority of LFD studies have been demonstrated to reach this threshold during the initial elimination phase, this has yet to be confirmed at long-term follow-up [66]. It is also worth noting that there may not be an optimal FODMAP threshold per se. Individuals with IBS may have differing degrees of visceral hypersensitivity, thereby not necessarily having symptoms triggered at a specific FODMAP threshold [67]. Individuals may therefore experience symptoms at different thresholds of FODMAP intake.

The majority of studies assessing the LFD have been dietetic-led, as well as being performed in secondary and tertiary care in an adult population. A study evaluating the long-term effect of dietetic-led interventions for IBS delivered in primary care has demonstrated satisfactory relief of gut symptoms at long-term follow-up (55%) [68]. However, further studies evaluating the use of a LFD, both physician-led and in primary care are required. In addition, the majority of studies have been performed in an adult population, with its applicability in a paediatric population requiring further exploration [69].

## Comparative efficacy of dietary therapies

There have been a number of RCTs comparing the LFD to TDA, with conflicting outcomes (Table 3). A RCT in Sweden demonstrated no significant difference in symptom improvement following the LFD and TDA at 4 weeks (50% vs 46%, *p* = 0.72) [48]. Likewise, a study in the US demonstrated no difference in adequate relief of symptoms in patients with IBS-D between individuals on LFD vs mNICE diet at 4 weeks (52% vs 41%, *p* = 0.31) [49]. The mNICE diet involved eating small frequent meals, avoiding trigger foods, and avoiding excess alcohol and caffeine. Foods containing FODMAPs were not specifically excluded [49]. In contrast a study in Iran demonstrated greater symptom improvement for individuals following a LFD in comparison to general dietary advice at 6 weeks [50]. A recent RCT also demonstrated a significantly higher proportion of responders on the LFD compared to TDA at both 4 weeks (63% vs 41%, *p* = 0.04), as well as 16 weeks (53% vs 31%, *p* = 0.03) [52]. This highlights the current uncertainty in terms of comparative efficacy between the LFD and TDA. A network meta-analysis ranked the LFD first amongst dietary therapies for global symptoms of IBS, above TDA [44]. Currently, it is unclear from the literature whether either dietary intervention is superior in the management of IBS.

**Table 3:** Key studies evaluating dietary therapies head to head

Lead author	Year	Study design	Study duration	Total number of participants	Comparator diets	Outcome
Bohn [48]	2015	RCT	4 weeks	75 IBS patients (Rome III)	TDA and LFD	No difference in clinical responders between TDA and LFD (50% vs 46%, *p* = 0.72)
Eswaran [49]	2016	RCT	4 weeks	92 IBS-D patients (Rome III)	mNICE and LFD	No difference in adequate symptom relief between mNICE and LFD (41% vs 52%, *p* = 0.31)
Zahedi [50]	2017	RCT	6 weeks	110 IBS-D patients (Rome III)	General dietary advice and LFD	LFD significantly improved overall gastrointestinal symptom scores, stool frequency and consistency compared to generalised dietary advice (*p* < 0.001, *p* < 0.001 and *p* = 0.003, respectively)
Paduano [23]	2019	Prospective study	4 weeks	42 IBS patients (Rome IV)	LFD, GFD and Mediterranean diet	LFD, GFD and Mediterranean diet showed the same efficacy in reducing disease severity (*p* < 0.01)
Goyal [52]	2021	RCT	16 weeks	101 IBS-D patients (Rome IV)	TDA and LFD	Higher proportion of responders on LFD compared to TDA at both week 4 (63% vs 41%, *p* = 0.0448) and week 16 (53% vs 31%, *p* = 0.0274)
Rej [20]	2022	RCT	4 weeks	101 IBS patients (Rome IV)	TDA, LFD and GFD	No difference in clinical response between TDA, LFD and GFD (42% vs 55% vs 58%, *p* = 0.43)

Total number of studies; *n* = 6, total number of participants; *n* = 521

There has only been one RCT to date comparing the efficacy of TDA, LFD and GFD head-to-head in a UK population [20]. This RCT in the UK demonstrated similar efficacy between all three diets, with no difference in clinical response rate at 4 weeks (42% TDA, 55% LFD, 58% GFD, *p* = 0.43) [20]. Similarly, a study in Italy showed comparative efficacy of LFD, GFD and Mediterranean diet [23]. However, the Mediterranean diet is not comparable with TDA. Currently, there is a lack of data evaluating the comparative efficacy of TDA, GFD and LFD at long-term follow-up, with studies needed.

Whilst comparable efficacy has been shown between these dietary therapies, differences in dietary acceptability have been noted in the short-term. TDA has been shown to be cheaper, less time-consuming to shop and easier to follow when eating out with family and friends in comparison to the GFD and LFD [20]. There have been no studies to date evaluating the acceptability of TDA and a GFD at long-term follow-up. However, long-term follow-up of patients on the LFD has demonstrated that it is significantly more expensive than a habitual diet and takes extra time to shop for, as well as negatively affecting social eating [55, 58]. In addition, patient preference may favour less complex diets, with a study in Italy demonstrating 86% would wish to continue on a Mediterranean diet, in comparison to 11% for a GFD and only 3% for a LFD [23].

The efficacy of dietary therapies compared to other treatments in IBS, including drug and psychological therapies, remains unclear. One RCT randomised participants to receive either hypnotherapy, dietary management or a combination and noted no significant difference in overall symptom improvement between groups (*p* = 0.67) [54]. Similarly, a RCT comparing yoga to the LFD failed to show differences in absolute IBS-SSS at either 12 (*p* = 0.151) or 24 weeks (*p* = 0.081) [70]. A meta-analysis of pharmacological trials in IBS demonstrated a pooled placebo response rate of 27% amongst the 73 RCTs included, using the global symptom response rate. It is likely that placebo effects are relevant to dietary studies also, which may impact assessment of dietary efficacy at short-term follow-up in particular, as placebo effects are known to wane with time [71].

In addition, the role of sucrose-isomaltase deficiency in IBS requires further exploration. The sucrase-isomaltase enzyme facilitates digestion of starch and sucrose. Whilst congenital sucrase-isomaltase deficiency (CSID) is rare, CSID mutations with known defective disaccharidase properties have been shown to be more frequent in IBS [72, 73]. Sucrase-isomaltase (SI) gene variants coding for disaccharidases with defective or reduced enzymatic activity have been shown to predispose to IBS [74], with a common SI variant (15Phe) being strongly associated [72, 75]. It has been suggested that the LFD may have a lower efficacy in individuals with reduced SI activity [76, 77]. Sucrose, and in part, starch are not specifically restricted as a part of a LFD, and therefore, the role of a low sucrose and low starch diet or enzymatic supplementation requires further exploration in IBS [77], in particular for individuals carrying hypomorphic SI variants [78].

## Conclusions

Currently, comparable efficacy has been demonstrated with TDA, GFD and LFD at short-term follow-up [20]. However, TDA appears to be more acceptable, in comparison to the GFD and LFD [20]. In view of this, trialling the TDA would be an appropriate first line dietary approach in IBS, consistent with current UK guidelines [7, 8]. However, a significant proportion of individuals note wheat to be a trigger in IBS [13]. In individuals who note gluten to be a primary trigger in IBS, a GFD may be more appropriate as a ‘bottom-up’ approach to manage symptoms [65]. Likewise, in individuals who wish to have a stricter reduction of FODMAPs, a LFD may be more appropriate, after dietary consultation.

The key to implementation of dietary therapies in IBS is to provide patient choice, in conjunction with dietetic assessment and advice. Ideally dietary therapies should be implemented by a dietitian, to prevent nutritional inadequacy, as the evidence base for dietary therapies has been derived from dietetic-led implementation of these therapies. Gastrointestinal-specific symptom anxiety, the fear of symptoms and consequence of this is a potential driver of food avoidance in IBS. This can potentially lead to disordered eating patterns in these patients, re-enforcing the vital role of dietetic involvement [79].

**Fig. 1:**
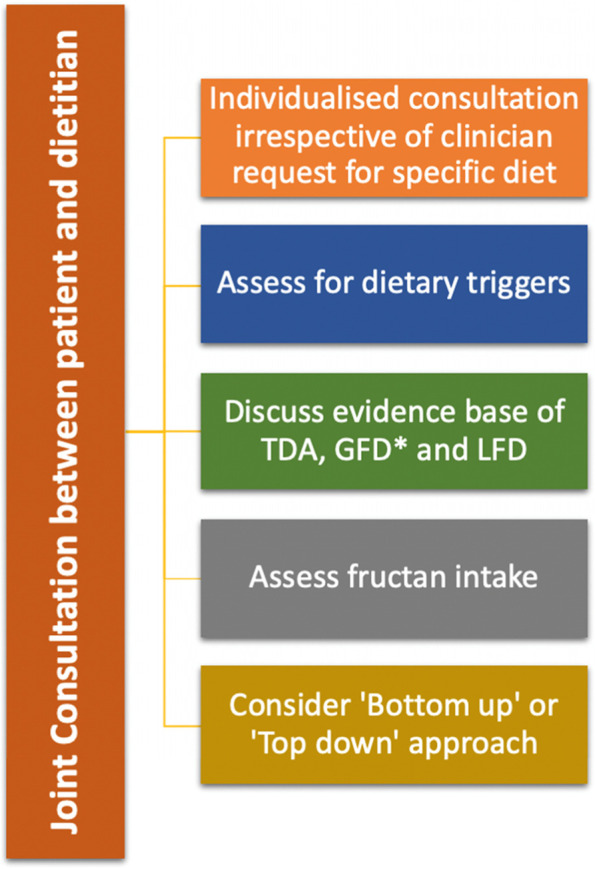
An individualised approach towards dietary therapies in IBS. TDA; traditional dietary advice, GFD; gluten-free diet, LFD; low FODMAP diet. Asterisk (*) symbol indicates the following: consider use of antigliadin antibodies as an adjuvant for selection to GFD (if available)

The majority of dietary advice given currently is physician-led, with only a minority of gastroenterologists referring to registered dietitians for IBS management [60]. In addition, there is a lack of dietitians available to deliver dietetic therapies, with a recent UK study highlighting an inequity of dietetic services across England [80]. In view of this, further research is required to assess the efficacy of a physician led approach in IBS. In addition, although novel methods of dietetic delivery, such as group sessions and webinars require further assessment, they may offer a more efficient method for delivering dietary therapies with scare resources [80–82]. There appears to be evidence for the use of dietetic therapies (TDA, LFD and GFD) to manage patients with IBS at short-term follow-up, with further research required on assessing the long-term efficacy of these approaches. Currently, the comparative efficacy of dietary therapies remains unclear due to a lack of head-to-head trials, and the current evidence fails to show superiority of one approach. The choice of dietary therapy should be tailored to the patient, in conjunction with a dietitian (Fig. 1). More research is required on the comparative efficacy of dietary therapies to non-dietary therapies.

## Data Availability

Not applicable.

## Abbreviations

ATI: Alpha-1 amylase trypsin inhibitor
CD: Coeliac disease
CSID: Congenital sucrase-isomaltase deficiency
FODMAPs: Fermentable oligo-, di-, mono- saccharides and polyols
GFD: Gluten-free diet
GI: Gastrointestinal
GSRS: Gastrointestinal Symptom Rating Scale
IBS: Irritable bowel syndrome
IBS-SSS: IBS Symptom Severity Score
LFD: Low FODMAP diet
MeSH: Medical subject headings
mNICE: Modified National Institute for Health and Care Excellence
RCT: Randomised controlled trial
SI: Sucrase-isomaltase
TDA: Traditional dietary advice
WGA: Wheat germ agglutinin
UK: United Kingdom
US: United States
